# Wishes, beliefs, and jealousy: use of mental state terms in *Cinderella* retells after traumatic brain injury

**DOI:** 10.3389/fnhum.2024.1386227

**Published:** 2024-05-14

**Authors:** Kathryn J. Greenslade, Cynthia Honan, Lauren Harrington, Laura Kenealy, Amy E. Ramage, Elise Bogart

**Affiliations:** ^1^Department of Communication Sciences and Disorders, University of New Hampshire, Durham, NH, United States; ^2^School of Medicine, University of Tasmania, Hobart, TAS, Australia; ^3^Interdisciplinary Program in Neuroscience and Behavior, University of New Hampshire, Durham, NH, United States; ^4^Faculty of Medicine and Health, The University of Sydney, Sydney, NSW, Australia

**Keywords:** social cognition, mental state terms, discourse, narration, traumatic brain injury

## Abstract

**Introduction:**

Traumatic brain injury (TBI) negatively impacts social communication in part due to social cognitive difficulties, which may include reduced mental state term (MST) use in some discourse genres. As social cognitive difficulties can negatively impact relationships, employment, and meaningful everyday activities, assessing and treating these difficulties post-TBI is crucial. To address knowledge gaps, the present study examined MST use in the narrative retells of adults with and without severe TBI to compare between-group performance, evaluate changes over the first two years post-TBI, and investigate the impact of participant and injury-related variables.

**Methods:**

The total number of MSTs, ratio of MSTs to total utterances, and diversity of MSTs were identified in the Cinderella narratives of 57 participants with no brain injury and 57 with TBI at 3, 6, 9, 12, and 24-months post-TBI.

**Results:**

Reduced MST use in participants with TBI was found at 3, 6, 9, and 12-months post-TBI, but these reductions disappeared when story length (total utterances) was accounted for. Further, MST diversity did not differ between groups. Similarly, although the total number of MSTs increased over time post-TBI, no changes were observed in the ratio of MSTs to total utterances or MST diversity over time. Injury severity (post-traumatic amnesia duration), years of education, and verbal reasoning abilities were all related to MST use.

**Discussion:**

Overall, although individuals used fewer MSTs in complex story retells across the first year following severe TBI, this reduction reflected impoverished story content, rather than the use of a lower ratio of MSTs. Further, key prognostic factors related to MST use included injury severity, educational attainment, and verbal reasoning ability. These findings have important implications for social communication assessment and treatment targeting social cognition post-TBI.

## 1 Introduction

People who survive a moderate-to-severe traumatic brain injury (TBI) often face hurdles in using social communication appropriately in everyday interactions (MacDonald, 2017; Keegan et al., 2023; Togher et al., 2023b). One factor contributing to these social communication challenges may be reduced social cognition, or the ability to infer other’s mental/emotional states, use these inferences to predict behavior, engage in social problem solving, and respond appropriately in social interactions (Turkstra, 2008; Spikman et al., 2012; McDonald, 2013; Bosco et al., 2017; Togher et al., 2023b). Social cognitive difficulties negatively impact a range of psychosocial outcomes, such as employment and community reintegration (Yeates et al., 2016; Westerhof-Evers et al., 2019). Thus, assessing and treating these difficulties post-TBI is critical to improving outcomes.

Social cognition is a multifaceted construct referring to cognitive processes that support perspective-taking in social interactions (Turkstra et al., 2017). To successfully see others’ points of view, people rely on their recognition of others’ emotions as well as their theory of mind (ToM) (Turkstra et al., 2017), or their ability to infer the unobservable mental states of others (Premack and Woodruff, 1978). These mental states include others’ knowledge, beliefs, guesses, plans, doubts, desires, emotions, etc. (Premack and Woodruff, 1978).

TBI can disrupt a range of social cognitive abilities, including recognizing emotions based on others’ facial expressions (Babbage et al., 2011; May et al., 2017) and prosody (McDonald, 2013), matching emotions to associated situations (Milders et al., 2003), inferring emotional states based on the eye region of human faces (Havet-Thomassin et al., 2006; Henry et al., 2006; Turkstra, 2008; Geraci et al., 2010; Muller et al., 2010), detecting inappropriate social behaviors (or faux pas) within stories (Milders et al., 2003; Geraci et al., 2010; Muller et al., 2010; May et al., 2017), and making social inferences based on video vignettes (Turkstra, 2008; McDonald et al., 2018; Theadom et al., 2019). Recently developed valid and reliable social cognitive assessments, such as the *Video Social Inference Test* (*VSIT*) (Turkstra, 2008), and *The Awareness of Social Inference Test* (*TASIT*) (McDonald et al., 2018), have enabled assessment of these abilities. However, the extent to which assessment performance aligns with everyday social communication is uncertain, as these assessments measure interpretation and understanding of social cues in hypothetical scenarios rather than in authentic interactions. Therefore, assessing social cognitive abilities in discourse may provide a more ecologically valid avenue for capturing these difficulties.

One method for assessing social cognition in discourse involves evaluating a speaker’s use of mental state terms (MSTs). MSTs are defined as words that represent the content of one’s own or others’ minds. These terms can be further categorized as desire terms (e.g., wish, hope, love), cognitive terms (e.g., believe, plan, see, beautiful, good), or emotional terms (e.g., jealous, upset) (Byom and Turkstra, 2012). In discourse, MSTs are used to share the perspective of the speaker, the communicative partner, or others (Bootsma et al., 2021). Thus, measuring the quantity of MSTs used within discourse (Armstrong, 2005; Armstrong and Ulatowska, 2007; Stronach and Turkstra, 2008; Armstrong et al., 2012; Byom and Turkstra, 2012, 2017; Bootsma et al., 2021; Myers et al., 2022) can provide a window into a speaker’s social inferences, their ability to make contextually appropriate adjustments (Byom and Turkstra, 2012), and the social acceptability of their discourse (Byom and Turkstra, 2017). Preliminary research suggests that MST use is a promising indicator of social cognition for adolescents and adults with TBI (Stronach and Turkstra, 2008; Byom and Turkstra, 2012, 2017). Yet, prior research also suggests that this approach’s utility varies depending on discourse type (e.g., conversation, narration), task features (e.g., level of intimacy, perspective-taking requirements), and participant characteristics (e.g., TBI severity, social cognitive abilities). For example, participants with moderate-to-severe TBI have shown no reduction in MST use within casual/low-intimacy conversations (Byom and Turkstra, 2012; Bootsma et al., 2021) or when they demonstrate better social cognitive abilities on the *VSIT* (Stronach and Turkstra, 2008). Adults with mild TBI have shown no reductions in their use of either cognitive process terms or terms with a negative emotional valence in narratives (Myers et al., 2022). In contrast, participants with moderate-to-severe TBI have been found to use significantly fewer MSTs per utterance when they had poorer social cognitive abilities (Stronach and Turkstra, 2008) and when they participated in either high-intimacy conversations (Byom and Turkstra, 2012) or conversations relying on perspective-taking abilities (Byom and Turkstra, 2017). Further, reductions in MST use when perspective-taking demands are high have been shown to relate to social acceptability ratings by naïve raters (Byom and Turkstra, 2017). Participants with TBI have also shown poorer adjustment in the type of MSTs used across intimacy levels, using more emotional MSTs in superficial as opposed to intimate conversations; participants with NBI showed the opposite pattern (Byom and Turkstra, 2012). Moreover, research suggests that communicative context (e.g., partner familiarity and skill) may affect conversational performance (Byom and Turkstra, 2012; Togher et al., 2023a); thus, such factors may also affect MST use in discourse.

Although rarely used in TBI research, in-depth analysis of MST use in narratives has proven informative in other clinical populations such as children with language disorders (Heilmann et al., 2010), adults with autism spectrum disorder (Rollins, 2014; Pham et al., 2023), and adults with aphasia (Armstrong, 2005). For example, the Narrative Scoring Scheme (NSS) (Heilmann et al., 2010) rates MST use, with a focus on how children describe *character/story-related* mental states (e.g., *Cinderella was sad*) rather than their own (e.g., *I cannot remember*, *I think…*). Thus, the Narrative Scoring Scheme captures children’s ability to infer and describe character/story-related mental states, reflecting social perspective-taking abilities. In applying the NSS score to adolescents and adults with autism spectrum disorder, Rollins (2014) found reduced use of MSTs to describe characters’ feelings and thoughts (lower NSS Mental State score) compared to controls; however, Pham et al. (2023) did not find this same reduction. In both cases, participants had average cognitive and general language abilities. Pham et al. (2023) reasoned that their participants might not have fully understood the intentions/roles of the MSTs they used, pointing toward potential comprehension challenges that were not measured by the narrative task. In adults with aphasia following stroke, Armstrong (2005) found that the ratio of mental state (cognitive and emotional) *verbs* to total verbs used in personal narratives did not differ between those with and without aphasia; however, adults with aphasia used more common/nonspecific mental verbs (e.g., know, think, want) and repeated the same verbs. A similar discrepancy in the use of specific compared to general terms has also been found when examining the broader category of evaluative language in personal narratives and conversation in those with aphasia (Armstrong and Ulatowska, 2007; Armstrong et al., 2012). Together, these findings suggest that the narratives of adults with aphasia may demonstrate a pattern of challenges with using a diverse variety of MSTs and using more specific terms. Such difficulties might indicate rote use of MSTs due to challenges related to lexical access or misunderstanding the underlying intentions of MSTs due to their abstractness or lack of imageability. The findings in these alternative clinical groups offer some insights into potentially useful approaches for MST evaluation in narrative discourse of individuals with moderate-to-severe TBI. Specifically, focusing on character/story-related MSTs, calculating a measure of MST diversity (e.g., dividing new MSTs by total MSTs to obtain an MST equivalent of a type-token ratio; MST-TTR), and performing a micro-analysis of specific vs. general MSTs could be important when analyzing MST use.

While recovery of MST use in discourse following TBI has not yet been examined, recent research has explored other aspects of discourse recovery over the first 12 to 24-months post-TBI (Elbourn et al., 2019a; Togher et al., 2023b; Greenslade et al., 2024). Elbourn et al. (2019a) showed improvements in the inclusion of accurate and complete story content in the *Cinderella* narratives of 57 adults with severe TBI between 3 and 6-months and between 9 and 12-months post-TBI. Tracking the same participant sample through 24-months post-TBI, Greenslade et al. (2024) identified improvements in story grammar organization, specifically in the inclusion of more story content (number of episodes, number of story-related propositions), the completeness of content (episode completeness), and the inclusion of non-essential “elaborated” elements (story elaboration) over the first 12-months post-TBI. Importantly, story elaboration was measured as the inclusion of multiple basic episodic elements (e.g., initiating events that introduce a problem/goal, attempts to solve the problem/attain the goal, direct consequences of the attempts), descriptive setting statements, and/or characters’ mental states. The observed improvements in elaboration, which may include descriptions of characters mental states (Greenslade et al., 2024), indicate that MST use may also improve over the course of recovery.

Interestingly, Elbourn et al. (2019a) and Greenslade et al. (2024) both found that while educational attainment was a protective factor, duration of post-traumatic amnesia (as an indicator of injury severity) was a risk factor in discourse recovery. Further examining such predictive relationships, Togher et al. (2023b) found that a measure of cognitive communication participation, the Functional Assessment of Verbal Reasoning and Executive Strategies (FAVRES) (MacDonald, 2005), at 6-months strongly predicted conversation participation at 24-months post-TBI. In contrast, Elbourn et al. (2019a) found that the presence of aphasia at 3-months following severe TBI was not related to discourse recovery over the first 12 months post-injury. While limited evidence currently exists about variables that influence discourse recovery, such information could contribute valuable insights for prognostic decision-making and resource allocation and should be a research priority (Togher et al., 2023b).

Overall, little is known about the nature of discourse tasks that are best suited to eliciting MSTs, how MST use may evolve over the first two years post-TBI, or what factors may be related to MST use post-TBI. To address these gaps in the literature and further refine the assessment of MSTs across discourse contexts, the present study compared MST use in the complex narrative retells of adults with and without severe TBI and investigated both trajectories of change over the first two years post-TBI and participant/injury-related variables that might relate to MST use post-TBI. Specifically, this study examined MST use in a complex fictional narrative (*Cinderella*) in healthy participants with no brain injury (NBI) and those with severe TBI at 3, 6, 9, 12, and 24-months post-injury. The *Cinderella* retell task was selected given its familiarity across Western cultures, its ability to be elicited and analyzed in a standardized manner, its complexity, and its strong potential for eliciting a wide variety of mental states (e.g., “her fairy godmother granted her wish,” “[no one could] believe how incredible she looked,” “her stepsisters, out of jealousy, destroy her dress”). These features were thought to be important as a less familiar or complex narrative might be less facilitative in eliciting speakers’ best performance in using MSTs. Expanding on prior research on social cognition in discourse, the current study’s coding for MST use focused on character/story-related mental states, rather than tangential/personal mental states (Heilmann et al., 2010), to explore perspective-taking challenges post-TBI (Byom and Turkstra, 2017). Further, an MST-TTR was calculated to determine MST diversity (vs. repeated use of the same terms). Finally, relationships between MST use and participant/injury-related variables were explored to identify factors other than general social cognitive abilities that may affect MST use post-TBI.

This study’s first research question asked whether participants with NBI would use more MSTs when retelling *Cinderella* compared to participants with severe TBI at 3, 6, 9, 12, and 24-months post-TBI. We hypothesized that early in recovery, participants with TBI would use fewer MSTs and/or less diversity of MSTs in their narratives than those with NBI. Given the mixed findings from prior research, no prediction was made about whether these group differences would persist into the later stages of recovery. The second research question asked whether MST use in participants with TBI would increase over the first two years post-TBI. We hypothesized that MST use would improve over the course of recovery post-TBI, with changes being more likely in the first 12-months (Elbourn et al., 2019a; Greenslade et al., 2024). The final research question asked whether demographic/injury-related variables (i.e., sex, age, educational attainment, length of post-traumatic amnesia, executive functioning/verbal reasoning, presence/absence of aphasia) would relate to MST use post-TBI. Based on prior findings of relationships between other narrative abilities and both educational attainment and injury severity (Elbourn et al., 2019a; Greenslade et al., 2024), similar relationships were anticipated with MST use. Further, following findings from Togher et al. (2023b), MST use was expected to correlate with executive functioning and verbal reasoning abilities on the FAVRES. Although the presence of aphasia is known to affect MST use following stroke (e.g., Armstrong, 2005), Elbourn et al. (2019a) found no differences in discourse trajectories post-TBI based on the presence of aphasia, using the same participant sample as the current study. Thus, no predictions were made about the effect that the presence of aphasia might have on MST use.

## 2 Materials and methods

### 2.1 Participants

The current study is a retrospective analysis of transcripts from the TBIBank Togher corpus (doi:10.21415/T5R018; Elbourn et al., 2019b), consisting of 57 Australian speakers with severe TBI, and 57 transcripts from the control database of AphasiaBank, consisting of US speakers with NBI, matched for age and educational attainment. Participants with TBI were part of a prospective cohort who contributed narratives at two or more of the following timepoints: 3, 6, 9, 12, and 24-months post-TBI. NBI participants, comprised of a cross-sectional cohort, contributed a single retell. Narrative transcripts were downloaded from the online, password protected TBIBank (Elbourn et al., 2019b) and AphasiaBank (MacWhinney et al., 2011) control databases. TBI transcripts were accessed from the Togher corpus (University of Sydney). As no Australian NBI transcripts were available, NBI transcripts of US speakers were accessed from control corpora contributed by four researchers (institutions): Capilouto (University of Kentucky), Boyle (Montclair State University), Richardson (University of New Mexico), and Wright (East Carolina University). Each contributing site obtained IRB approval to collect and share data with TBIBank or AphasiaBank for future research use; all participants provided written informed consent or assent with written informed consent of a designee.

Table 1 summarizes demographic characteristics of the TBI and NBI groups. Participants with TBI met the following inclusion criteria for the original study: post-traumatic amnesia (PTA; > 24 h) and/or Glasgow Coma Scale score^1^ (< 8) indicating severe TBI; chronological age between 16 and 65 years at the time of injury; participation starting after being medically stable with recovery from PTA; English language proficiency; and residence within a three-hour distance of Sydney, Australia. Exclusion criteria included an inability to obtain consent from the person with TBI or designee; prior history of neurological injury/illness or significant medical condition (e.g., developmental delay); persistent PTA; initial assessment being greater than 7 months post-TBI; and inability to collect at least one follow-up data point. Additional detail about participants with TBI is available in Elbourn et al. (2019a).

**Table 1:** Demographic characteristics of the TBI and NBI groups, with results of significance testing.

Demographic characteristic	TBI (*n* = 57)	NBI (*n* = 57)	χ^2^	*p* value	*w*
Sex (M:F)	46:11	35:22	5.16	0.038	0.213
Race/ethnicity	41 Oceanian (Non-indigenous) 4 North-West European 4 Central Asian 3 South-East Asian 2 “The Americas” 1 Sub Saharan African 1 North African/Middle Eastern 1 Oceanian (Indigenous)	53 Caucasian 2 African-American 2 Hispanic/Latino	N/A		
Language status^*^	46 Monolingual 8 Bilingual 3 Multilingual	35 Monolingual 3 Multilingual 19 NR	2.36	0.150	0.157
Primary language^*^	52 English 5 Other	56 English 1 NR	5.14	0.057	0.213

	Mean (SD) Range	Mean (SD) Range	*Z*	*p*	*d*
Age (years)^a^	35.25 (13.11) 16–66	35.61 (13.03) 18–66	−0.20	0.843	0.028
Educational attainment (years)	13.58 (2.99) 8–20	14.43 (1.54) 12–18	−1.84	0.065	0.357
PTA duration	52.88 (40.03) 6–215	–	–	–	–
GCS score	6.83 (3.47) 3–15	–	–	–	–
FAVRES total reasoning subskills raw score, 6-months post-TBI (*n* = 32)	62.53 (14.81) 31–89	–	–	–	–

M:F, Male to Female; NR, not reported; PTA, post-traumatic amnesia; GCS, Glasgow Coma Scale; FAVRES, Functional Assessment of Verbal Reasoning and Executive Strategies; TBI, traumatic brain injury; NBI, non-brain injured.^*^Statistic excludes “not reported” primary language/language status.

a TBI group: age at injury, NBI group: age at participation.

The NBI sample included 57 adults, who resided in the United States, spoke English as their primary language, reported adequate hearing/vision, and had no history of neurological injury/disease or speech/language disorder.

### 2.2 Procedure

*Cinderella* narratives were elicited using the internationally standardized TBIBank protocol (Elbourn et al., 2019b, 2023), developed in collaboration with researchers from multiple Western countries (United States, Canada, Australia, the United Kingdom). *Cinderella* was selected for the protocol as a prototype of an overlearned Western narrative structure, which is familiar across Western cultures – including the United States and Australia, where the current study’s speakers resided. Details about the *Cinderella* elicitation protocol are available here: https://tbi.talkbank.org/protocol/instructions-TBI.doc. After sample collection, each collecting site transcribed participants’ narratives, divided them into utterances (c-units, defined as an independent clause and all attached dependent clauses; Loban, 1976), and uploaded them to the TBIBank (TBI sample) or AphasiaBank control database (control sample). A limited number of transcripts were not readily available (*n* = 7, 8, and 33 at 6, 9, and 24-months, respectively) and thus were transcribed from the original video or audio samples, using the same CHAT transcription conventions.

### 2.3 Measures

#### 2.3.1 Coding MST use within a story grammar framework

Each transcript from TBIBank/AphasiaBank was first downloaded and then assigned a coding identification number, using a random number generator in Excel. *Cinderella* transcripts were extracted, and the EVAL command in CLAN was run to determine the total number of utterances in each transcript. Then, MST coding was completed without viewing videos. This approach allowed coders to be blind to participant diagnosis (TBI vs. NBI) and time point (for participants with TBI). However, between-group differences in narrative length, quality, and linguistic variations (e.g., more colloquial/informal lexical selection among Australian speakers compared to US speakers) could have impacted coding blindness.

Before coding for MST use, transcripts were pasted into an Excel spreadsheet and divided into propositions. Propositions are defined as a verb phrase (predicator) including all related arguments and generally correspond with clauses. In rare cases, propositions were divided if they served distinct purposes within a story (e.g., “At the strike of midnight, Cinderella ran away.” = ^1^At the strike of midnight [introduction of a new problem], ^2^Cinderella ran away [action addressing the problem]).

For each transcript, coders identified the number of MSTs used (“MST tokens”), the specific term(s) used, and the number of MSTs that had not previously been used in that transcript (i.e., the number of different MSTs used, or “MST types”) in each proposition. Excel formulas automatically summed the total number of MSTs and total number of different MSTs across propositions. To identify MSTs, coders referenced a list of MSTs compiled from the appendices of Bootsma et al. (2021) and Byom and Turkstra (2012) as well as the traditional story grammar coding manual from Greenslade et al. (2024). This compiled list is included in Supplementary material. When coders questioned whether a word should be considered an MST (e.g., *cranky*), an online dictionary definition (i.e., from Oxford Languages/Google; Merriam-Webster/others used for further clarification) was consulted to determine whether the definition used an identifiable MST (i.e., *ill-tempered*), or whether synonyms were MSTs (e.g., *irritable/grumpy*). Words determined to be MSTs based on coder consensus were added to the existing MST list, and coders reviewed prior transcripts to ensure that all instances of that term were identified. Following Heilmann et al. (2010), MSTs that referred to the speaker or listener (e.g., “I think,” “you know”) or were otherwise tangential to the story were identified and counted, but were excluded from current analyses as such utterances did not reflect speakers’ ability to take characters’ perspectives. Similarly, MSTs used within repetitions or revisions were identified but excluded to avoid overinflating scores for speakers who frequently needed to reformulate their thoughts. The total number of MSTs, the ratio of MSTs to the total utterances, and the ratio of different MSTs (MST types) to the total number of MSTs (MST tokens), or MST type-token ratio (hereafter MST-TTR), were dependent variables, indicating participants’ social cognitive abilities.

##### 2.3.1.1 Training and reliability

The first author trained three graduate student research assistants to divide transcripts into propositions as part of a larger project examining story grammar organization; reliability for this project is reported in Greenslade et al. (2024). Subsequently, one graduate (fourth author) and one undergraduate research assistant (third author) were trained to identify and count MSTs within pre-identified propositions. Training concluded after 14–20 h when the research assistants achieved point-to-point agreement of greater than 80% (81.82%) in identifying the same terms in a set of five transcripts. In the full data set, 79 of 295 transcripts (26.78%) were randomly selected for reliability analysis using a random number generator in Excel; point-to-point agreement for identifying the same terms was 84.61%. Further, agreement for identifying the occurrence/nonoccurrence of MST types and tokens in each proposition was found to be *Κ* = 0.95 in both cases.

#### 2.3.2 Executive functioning and verbal reasoning

The Functional Assessment of Verbal Reasoning and Executive Strategies (FAVRES; MacDonald, 2005) was delivered as a measure of executive functioning and verbal reasoning abilities at 6-months post-TBI. The FAVRES requires examinees to apply higher-level executive functioning abilities (e.g., planning, making decisions, solving problems) within four functional scenarios. The Total Accuracy, Total Rationale, and Total Reasoning subskills raw scores were used as dependent variables to examine the relationship between MST use in narratives and executive functioning/verbal reasoning abilities on a standardized assessment.

#### 2.3.3 Presence of aphasia

The Western Aphasia Battery (WAB; Kertesz, 2012) Aphasia Quotient was calculated as a measure of language abilities at 3-months post-TBI. Aphasia Quotient scores below 93.8 were considered to indicate the presence of aphasia. This dichotomous variable was used to determine whether the presence of aphasia affected MST use.

## 3 Data analysis

Using Statistical Package for the Social Sciences (SPSS v28), descriptive statistics, skew and kurtosis, and Shapiro–Wilk’s normality tests were generated for the total number of MSTs, ratio of MSTs to total utterances, and MST-TTR. All three variables were non-normally distributed based on Shapiro–Wilk’s test *p* values <0.05 (Laerd Statistics, n.d.). Thus, to answer the first research question regarding between-group differences, Mann–Whitney *U* tests were conducted to compare MST use in the TBI and NBI groups. For each variable, a more conservative alpha level of 0.01 was used to control for Type I error rate.

To examine trajectories of change in MST use over the first two years post-TBI as well as associations with demographic/injury-related factors, R and RStudio software were used to impute missing MST (dependent variable) data and construct generalized estimating equation (GEE) models. First, because approximately 20% of MST data were missing at random (but not completely at random), multiple imputation by chained equations (MICE) was used to impute the missing data. Next, GEE models with first-order autoregressive correlation structures were constructed for each dependent variable, using timepoint (3, 6, 9, 12, and 24 months) as a repeated measures factor. Demographic/injury-related factors (i.e., age, gender, years of education, PTA duration as an index of injury severity) were included as covariates; based on a correlation matrix, no multicollinearity was found between independent variables. Because the total number of MSTs was a count variable, the GEE model for this variable used a Poisson distribution. In contrast, the ratio of MSTs to total utterances and MST-TTR were ratio data; thus, the GEE model for these variables used a Gamma distribution with a log link function. Further, to address issues with taking the log of zero with ratio data, the minimum, non-zero value for each dependent variable was identified, divided by 100, and added as a constant to each value of that variable. Resulting coefficients were exponentiated to obtain incidence rate ratios (IRRs) for the Poisson model and multiplicative change factors (MCFs) for gamma with log link models. Both IRRs and MCFs were interpreted as the estimated rate of change in the dependent variable for every one unit increase in the independent variable, holding other variables constant. IRRs/MCFs less than 1 signaled lower dependent variable values; IRRs/MCFs greater than 1 signaled higher values. For each model, an alpha of 0.017 (0.05 divided by 3) was used to control for the three models.

Finally, given that only 31 participants with TBI had FAVRES scores at 6-months post-TBI, these scores were not entered into GEE models as covariates, as missingness in independent variables is not permitted. Instead, Spearman’s rank-order correlations were conducted between each FAVRES Total score and each MST variable. Similarly, only 31 participants with TBI had WAB Aphasia Quotient scores at 3-months post-TBI. Because Aphasia Quotient scores were dichotomized to indicate presence/absence of aphasia, Mann–Whitney U-tests were used to compare MST use in those with TBI who met criteria for aphasia and two groups: (1) their NBI matches and (2) those with TBI who did not meet criteria for aphasia. For each variable, a more conservative alpha level of 0.01 was used to control for Type I error rate.

## 4 Results

**Figure 1:**
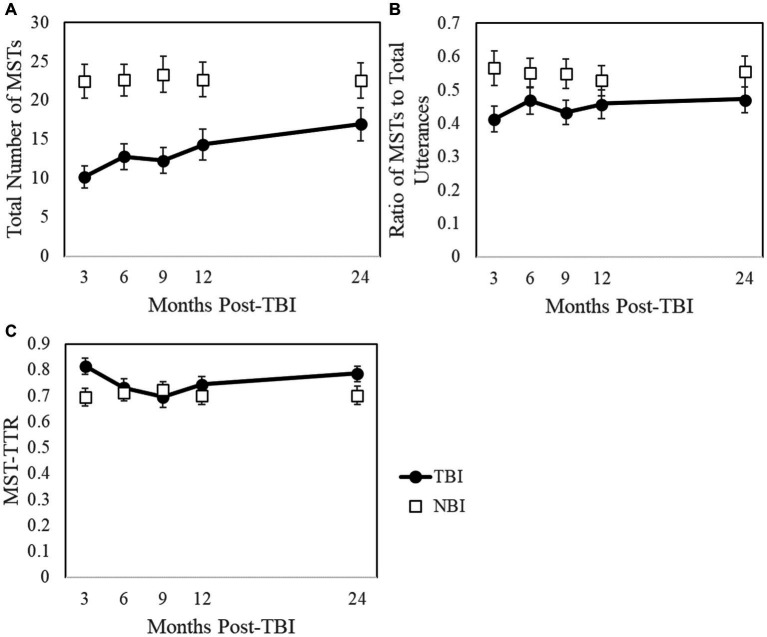
Trajectories across the first two years post-TBI with NBI comparisons for: **(A)** the total number of mental state terms (MSTs), **(B)** ratio of MSTs to total utterances, and **(C)** mental state term type-token ratio (MST-TTR). Because NBI data represent a single time point, they are not connected by a line. Given that not all participants with TBI contributed at each time point, and only NBI data with a TBI match were included, NBI averages vary across time points. Error bars are ±1 standard error.

Figure 1 illustrates comparisons between the TBI and NBI groups as well as trajectories across the first two years post-TBI for: (A) the total number of MSTs, (B) ratio of MSTs to total utterances, and (C) MST-TTR.

### 4.1 Between group differences

Table 2 summarizes descriptive and inferential statistics for the total number of MSTs, ratio of MSTs to total utterances, and MST-TTR for the TBI and NBI groups at each time point. Mann–Whitney U-tests revealed that at 3, 6, 9, and 12-months post-TBI, participants with TBI included fewer MSTs in Cinderella narratives than those with NBI. However, after controlling for story length (i.e., ratio of MSTs to total utterances), differences in MST use were no longer apparent at any timepoint. At 3-months post-TBI, the TBI group demonstrated higher MST-TTR values compared to the NBI group (*U* = 1294.00, *Z* = 2.74, *p* = 0.006, *r* = 0.292). Closer examination of the data revealed that six of 44 participants with TBI included only one MST, resulting in an inflated MST-TTR of 1 and accounting for this significant difference. Importantly, MST-TTR values at 3-months no longer differed when these six participants were excluded from analyses (*U* = 1039.00, *Z* = 1.89, *p* = 0.058, *r* = 0.201). No between-group differences in MST-TTR existed at later time points.

**Table 2:** Descriptive and inferential statistics comparing the TBI and NBI groups for variables measuring mental state term (MST) use at 3, 6, 9, 12, and 24-months post-TBI.

Dependent variable		Total number of MSTs							Ratio of MSTs to total utterances							MST-TTR						
Group		TBI		NBI		Comparison			TBI		NBI		Comparison			TBI		NBI		Comparison		
Time point	*n* per group	*M*	*(SD)*	*M*	*(SD)*	*Z*	*p*	*r*	*M*	*(SD)*	*M*	*(SD)*	*Z*	*p*	*r*	*M*	*(SD)*	*M*	*(SD)*	*Z*	*p*	*r*
3 m	44	10.18	(9.36)	22.45	(14.23)	−4.26	<0.001^*^	0.455	0.41	(0.25)	0.57	(0.34)	−2.04	0.041	0.218	0.81	(0.21)	0.69	(0.23)	2.74	0.006^*^	0.292
6 m	53	12.79	(12.09)	22.62	(14.68)	−3.88	<0.001^*^	0.376	0.47	(0.30)	0.55	(0.32)	−1.18	0.239	0.114	0.73	(0.25)	0.71	(0.22)	1.10	0.274	0.106
9 m	44	12.27	(11.13)	23.34	(15.14)	−3.92	<0.001^*^	0.418	0.43	(0.24)	0.55	(0.29)	−1.47	0.143	0.156	0.69	(0.27)	0.72	(0.21)	−0.09	0.930	0.009
12 m	46	14.30	(13.39)	22.67	(15.13)	−3.04	0.002^*^	0.317	0.46	(0.29)	0.53	(0.30)	−1.12	0.264	0.116	0.74	(0.21)	0.70	(0.23)	1.12	0.262	0.117
24 m	43	16.93	(14.18)	22.56	(15.05)	−1.98	0.048	0.213	0.47	(0.25)	0.55	(0.31)	−1.14	0.254	0.123	0.78	(0.19)	0.70	(0.24)	1.81	0.071	0.195

TBI, traumatic brain injury; NBI, no brain injury; MST, mental state term; MST-TTR, mental state term type-token ratio (number of new MSTs divided by total MSTs); m, months post-TBI.^*^ Significant at or below 0.01 (adjusted alpha). Given that not all participants with TBI contributed at each time point, and only NBI data with a TBI match were included, NBI averages vary across time points.

### 4.2 Trajectories of MST use over the first two years post-TBI

Table 3 summarizes the results of the GEE models examining the effects of time, years of education, duration of PTA, gender, and age. In the TBI group, GEE models investigated within-subject changes in MST use between 3 and 24-months post-TBI, using timepoint as a repeated measures factor and covarying for demographic/injury-related factors. The GEE model for the total number of MSTs revealed that for each one-unit increase in time (e.g., change from 3-month to 6-month timepoint), the expected rate of increase was 1.082 for the total number of MSTs, representing a statistically significant improvement over time (*p* = 0.002). However, no significant changes over time were detected when controlling for story length, ratio of MSTs to total utterances: *p* = 0.076, or when evaluating the diversity of MSTs, MST-TTR: *p* = 0.960.

**Table 3:** Results of generalized estimating equation (GEE) models, including effects of time and demographic/injury-related covariates (i.e., years of education, post-traumatic amnesia [PTA], gender, age).

Variable	B	SE	IRRs^†^/ MCFs^††^	95% CI	*p*-value
Model 1: Total number of MSTs					
Time	0.079	0.377	1.083	[1.028, 1.139]	0.002^*^
Years of education	0.123	0.020	1.131	[1.087, 1.176]	<0.001^*^
PTA	−0.005	0.002	0.995	[0.991, 0.998]	0.002^*^
Gender	−0.250	0.026	0.779	[0.599, 1.012]	0.061
Age	0.006	0.005	1.006	[0.996, 1.105]	0.225
Model 2: Ratio of MSTs to total utterances					
Time	0.035	0.187	1.036	[0.996, 1.077]	0.076
Years of education	0.066	0.014	1.068	[1.039, 1.098]	<0.001^*^
PTA	−0.004	0.001	0.996	[0.993, 0.999]	0.005^*^
Gender	−0.038	0.097	0.962	[0.795, 1.165]	0.694
Age	0.004	0.004	1.004	[0.996, 1.012]	0.325
Model 3: MST-TTR					
Time	−0.001	0.016	0.999	[0.612, 1.070]	0.960
Years of education	0.007	0.008	1.007	[0.991, 1.024]	0.368
PTA	−0.003	0.001	0.997	[0.996, 0.999]	0.001^*^
Gender	−0.045	0.049	0.956	[0.869, 1.051]	0.353
Age	−0.0005	0.002	1.000	[0.996, 1.003]	0.780

MST, mental state term; MST-TTR, mental state term—type-token ratio; PTA, duration of post-traumatic amnesia in days; IRR, Incident Rate Ratio; MCF, Multiplicative Change Factors. ^*^Significant at or below 0.017 to account for 3 models. ^†^Model 1,^††^Models 2 and 3.

### 4.3 Associations between MST use and demographic/injury-related factors

While improvements in MST use over time were not found after controlling for story length, the GEE models did identify demographic/injury-related variables that influenced MST use. Specifically, holding all other independent variables constant, each one-day increase in PTA predicted a decrease in the total number of MSTs (IRR = 0.995, *p* = 0.002), ratio of MSTs to total utterances (MCF = 0.996, *p* = 0.005), and MST-TTR (MCF = 0.997; *p* = 0.001), indicating the inclusion of fewer MSTs or less diversity of MSTs. In contrast, each additional year of education predicted an increase in the total number of MSTs (IRR = 1.131, *p* < 0.001) and ratio of MSTs to total utterances (MCF = 1.068, *p* < 0.001), indicating the inclusion of more MSTs. Educational attainment was not a predictor for diversity of MSTs (MST-TTR). Further, age and gender did not have a statistically significant impact on any MST variable.

**Figure 2:**
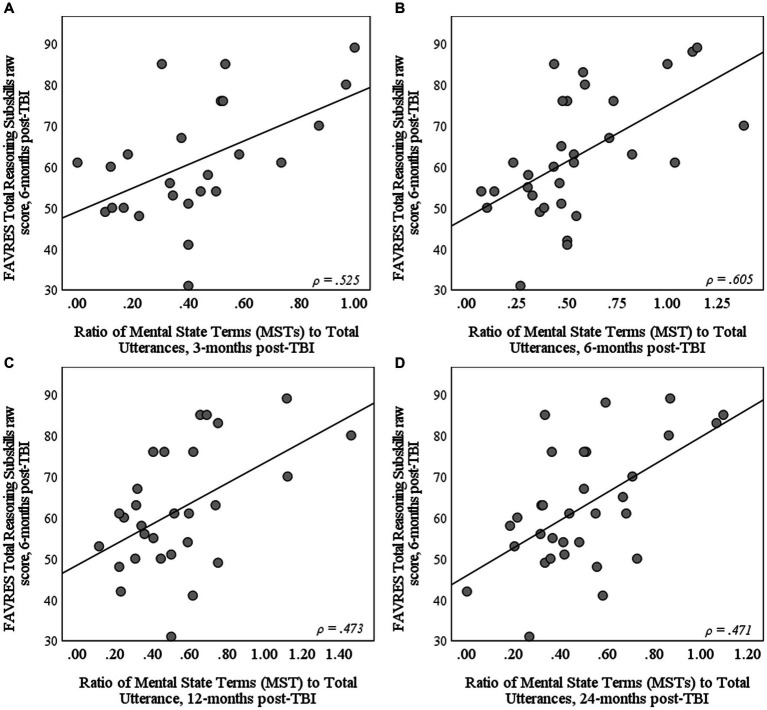
Scatterplots depicting the relationship between FAVRES Total Reasoning Subskills raw score at 6-months post-TBI and the ratio of mental state terms (MSTs) to total utterances at **(A)** 3, **(B)** 6, **(C)** 12, and **(D)** 24-months post-TBI.

Table 4 summarizes Spearman’s rank-order correlations results for relationships between the three MST use variables and the three FAVRES raw scores. Results revealed moderate relationships between FAVRES Total Reasoning Subskills scores at 6-months and the total number of MSTs at all timepoints post-TBI. After controlling for story length (ratio of MSTs to total utterances), moderate correlations were still found between 6-month reasoning scores and MST use at all timepoints post-TBI (see Figure 2), except for 9-months (*ρ* = 0.457, *p* = 0.015). Finally, moderate inverse correlations were found between 6-month reasoning scores and MST-TTR values at 3-months. Of note, correlations with MST-TTR values at 3-months remained significant, even after removing the six TBI participants who used a single MST in their *Cinderella* retells (*ρ* = −0.523, *p* = 0.010). In contrast to relationships with verbal reasoning scores, the only significant correlation with Total Rationale scores was with the ratio of MSTs to total utterances at 6-months post-TBI; Total Accuracy scores were not significantly correlated with any MST use variable at any timepoint.

**Table 4:** Spearman rank-order correlations between MST use variables and executive functioning/ verbal reasoning scores.

MST use variable/timepoint	FAVRES total accuracy, raw score		FAVRES total rationale, raw score		FAVRES total reasoning subskills, raw score	
	*ρ*	*p*	*ρ*	*p*	*ρ*	*p*
Total number of MSTs						
3 m (*n* = 25)	0.482	0.015	0.306	0.136	0.690	<0.001^**^
6 m (*n* = 32)	0.396	0.025	0.419	0.017	0.682	<0.001^**^
9 m (*n* = 28)	0.292	0.132	0.397	0.037	0.724	<0.001^**^
12 m (*n* = 29)	0.310	0.102	0.406	0.029	0.779	<0.001^**^
24 m (*n* = 32)	0.307	0.087	0.153	0.404	0.664	<0.001^**^
Ratio of MSTs to total utterances						
3 m (*n* = 25)	0.152	0.469	0.042	0.841	0.525	0.007^*^
6 m (*n* = 32)	0.352	0.048	0.458	0.008^*^	0.605	<0.001^**^
9 m (*n* = 28)	0.125	0.525	0.273	0.160	0.457	0.015
12 m (*n* = 29)	0.325	0.086	0.451	0.014	0.473	0.009^*^
24 m (*n* = 32)	0.290	0.107	0.042	0.821	0.471	0.006^*^
MST-TTR						
3 m (*n* = 25)	−0.453	0.023	−0.370	0.068	−0.574	0.003^*^
6 m (*n* = 32)	−0.297	0.098	−0.397	0.024	−0.254	0.161
9 m (*n* = 28)	0.261	0.180	0.314	0.104	0.050	0.801
12 m (*n* = 29)	−0.192	0.318	−0.233	0.224	−0.402	0.030
24 m (*n* = 32)	0.088	0.633	0.343	0.055	−0.141	0.443

MST, mental state term; FAVRES: Functional Assessment of Verbal Reasoning and Executive Strategies (all scores from 6-month time point); m, months; MST-TTR, mental state term—type-token ratio. An adjusted alpha of 0.01 was set for all correlations.^*^p < 0.01, ^**^ *p* < 0.001.

As with differences between the full TBI and NBI samples, Mann–Whitney U-tests revealed a significant difference between MST-TTRs in participants with aphasia and their NBI matches at 3-months (*U* = 46.00, *Z* = 2.79, *p* = 0.005, *r* = 0.509); however, upon removal of three participants with TBI who used only one MST, differences were no longer significant based on the adjusted alpha level (*U* = 31.50, *Z* = 2.55, *p* = 0.011, *r* = 0.510). No other significant differences between either participants with TBI who met criteria for aphasia and their NBI matches, or participants with TBI who did or did not meet criteria for aphasia (*p* > 0.05) were found at any other timepoint for total number of MSTs, ratio of MSTs to total utterances, or MST-TTR.

## 5 Discussion

At face value, results addressing this study’s first two research questions suggested that individuals with severe TBI used fewer MSTs than individuals with NBI and demonstrated increased use of MSTs in their complex story retells over the first two years post-TBI. However, both the between-group differences and changes over time post-TBI were more apparent than real, as both could be attributed to participants with TBI telling shorter stories that included less content in the sub-acute stage of recovery, as described in Greenslade et al. (2024), rather than using a lower ratio of MSTs to total utterances. Using the same sample of participants as the current study, Greenslade et al. (2024) found that participants with TBI increased the amount of content included in their *Cinderella* retells as evidenced by an increasing number of episodes between 3 and 6-months post-TBI as well as more story-related propositions between 3 and 9-months post-TBI.

The current study indicates that as participants included this additional content in their stories, MST use increased at a proportionate rate. Yet, it is important to acknowledge that although the rate and diversity of MST use may be comparable to NBI counterparts, reduced content may lead communication partners to perceive the discourse of individuals with TBI as being less socially acceptable or reflecting poorer social cognition due to the lower total number of MSTs used, as observed in Byom and Turkstra (2017). Thus, the results highlight the impact that impoverished discourse of individuals with severe TBI may have on social communicative exchanges and reinforces impoverished discourse as a chronic issue that could benefit from long-term support.

Related to our third research question, three demographic/injury-related factors influenced MST use in narratives. Specifically, participants with TBI who had higher levels of education included a larger number and ratio of MSTs in their *Cinderella* retells as compared to those with lower education levels. Thus, educational attainment may be a protective factor for MST use, as it is for discourse changes over time post-TBI more broadly (Elbourn et al., 2019a; Greenslade et al., 2024), consistent with cognitive reserve theory (Kesler et al., 2003; Steward et al., 2018). On the other hand, participants who experienced more days of PTA following their TBI tended to include a smaller number and ratio of MSTs compared to those with fewer days of PTA. Thus, PTA duration is likely a risk factor for MST use, as it is for discourse changes over time post-TBI more broadly (Elbourn et al., 2019a; Greenslade et al., 2024). Because PTA duration corresponds with injury severity, this finding suggests that milder injuries may not be associated with social cognitive difficulties on this measure, whereas more severe TBIs are associated with lower rates of MST use. This is consistent with the existing research literature (Stronach and Turkstra, 2008; Byom and Turkstra, 2012, 2017; Myers et al., 2022). Further, verbal reasoning abilities (FAVRES scores) at 6-months post-TBI were significantly related to the number and ratio of MST use at each timepoint. This finding suggests that either challenges with verbal reasoning are related to social cognitive deficits post-TBI, or common neural mechanisms lead to deficits in both domains. This relationship was logical given that the FAVRES Total Reasoning Subskills score relates to the speaker’s ability to identify which information is most/least important, provide advantages and disadvantages for different options (not just their own selection), consider different choices and potential changes to their choice if new information is provided, and predict consequences of certain choices. Thus, the Total Verbal Reasoning Subskills score reflects speakers’ use of abstract verbal language to make judgements and draw conclusions, likely requiring the use of MSTs. In contrast, neither participants’ accuracy nor their rationales in responding to executive functioning tasks on the FAVRES were related to MST use. Overall, these findings align with prior research that suggests (1) early verbal reasoning scores on the FAVRES are prognostic of later communication outcomes (Togher et al., 2023b), and (2) only individuals post-TBI who show social cognitive (Stronach and Turkstra, 2008) or verbal reasoning deficits show reduced MST use. The latter effect may be particularly apparent on discourse tasks that necessitate perspective-taking, such as high-intimacy conversations (Byom and Turkstra, 2012) or persuasion (Byom and Turkstra, 2017). Importantly, the diversity of MST use at two timepoints (3 and 12-months post-TBI) was inversely related to verbal reasoning abilities. This suggests that although social cognition and verbal reasoning abilities may share some common resources/neural pathways, social cognition as measured by MST diversity may rely on distinct resources/neural pathways and contribute uniquely to the social profile of individuals following a severe TBI. Finally, no associations were found between the presence of aphasia and MST use in the present study; this result was consistent with Elbourn et al.’s (2019a) finding that in the same sample of participants, the presence of acute aphasia at 3-months did not alter trajectories of discourse changes over time post-TBI. Understanding these and other prognostic factors is critical to helping clinicians and researchers predict outcomes and identify individuals who may need more support for poor social cognition.

### 5.1 Implications for discourse analysis and treatment in adults with TBI

The importance of social cognition in everyday life should not be underestimated. Impaired social cognition can negatively impact a speaker’s ability to effectively infer and/or convey intentions, use inferences to predict behavior, engage in social problem-solving, take others’ perspectives, judge social closeness, and maintain an appropriate degree of intimacy with people who vary in their familiarity (Turkstra, 2008; Shorland and Douglas, 2010; Byom and Turkstra, 2012; Spikman et al., 2012; McDonald, 2013; Bosco et al., 2017). Such challenges impact an individual’s ability to engage appropriately in interactions with work colleagues, friends, and others in the community (Shorland and Douglas, 2010; Yeates et al., 2016; Westerhof-Evers et al., 2019). In turn, compromised relationships can lead to dwindling support networks, feelings of isolation, and negative repercussions for mental/emotional health and wellbeing (Douglas, 2020). In contrast, when treatment explicitly addresses social cognition, improvements may be observed in relationship quality, role resumption, attainment of treatment goals, and quality of life/life satisfaction (Westerhof-Evers et al., 2017; Lohaus et al., 2024). Thus, the current study’s findings offer insights for clinical assessment and treatment post-TBI, which may have meaningful impacts for individuals with TBI.

The current study found that individuals with severe TBI produced an appropriate ratio and variety of MSTs in their narrative retells over the first two years post-injury. Importantly, this study used a fictional narrative with predictable/familiar structures and content that had low personal relevance; further, storytelling was preceded by visual stimuli to support memory. Thus, these discourse features may offer critical supports to facilitate MST use. However, alternate interpretations are possible. First, the familiarity of *Cinderella* may have led to the rote or automatic use of MSTs (e.g., Cinderella is very beautiful), meaning participants might not have fully understood the judgements/intentions underlying the terms they used (as suggested in Pham et al., 2023). Thus, to fully capture a person’s social cognitive capabilities post-TBI, the comprehension of MSTs and other metacognitive language may need to be measured rather than only assessing production (Grazzani and Ornaghi, 2012). Alternately, part of the challenge with assessing MSTs in the early stages post-TBI might be identifying a task that elicits sufficient language to reveal deficits. In the subacute stage of recovery, this study’s participants with TBI tended to generate a small total number of utterances; thus, they produced fewer MSTs, despite producing a similar ratio of MSTs to total utterances. It is possible that if a longer sample were elicited at this stage, the associated demands (e.g., attention, working memory, executive functioning) might stress the system enough to reveal social cognitive deficits. Shorland and Douglas (2010) hinted at such an intersection between attention and social cognition in their report of interviews with two adults with TBI; specifically, interviewees noted that it was more challenging to feign interest, use body language appropriately, and participate socially when fatigued. Further, other personal and partner factors may affect performance, including pre-injury factors (e.g., education, communication style), self-regulation/control functions (e.g., self-appraisal), familiarity/closeness of partners, and more (MacDonald, 2017; Keegan et al., 2023). Such performance variations across different contexts and demands are critical to consider in TBI, as illustrated in both MacDonald (2017) and Keegan et al.’s (2023) models of social communication.

Situating the current study’s findings in the existing literature (Byom and Turkstra, 2012, 2017; Bootsma et al., 2021), an assessment battery may be needed to capture the range of social cognitive abilities displayed post-TBI, similar to recommendations for a social communication battery by Sohlberg et al. (2019). To assess social cognition via MST use in discourse, tasks will need to be carefully designed to account for features that facilitate better performance or capture challenges. Specifically, facilitators of MST use may include the provision of visual stimuli to support memory and the elicitation of familiar (overlearned) content that does not evoke personal emotions (i.e., low intimacy/personal relevance; Byom and Turkstra, 2012; Bootsma et al., 2021). In contrast, to capture social cognitive challenges based on MST use, discourse tasks appear to need to sufficiently ‘stress the system’ by capturing a longer sample (i.e., to increase cognitive demands and expectations for the use of more MSTs), focusing on personally relevant content (i.e., high intimacy/ emotional investment; Byom and Turkstra, 2012) and/or taxing executive functioning/working memory (e.g., not providing visual stimuli; rule of not saying “the” as described by Byom and Turkstra, 2017). Thus, to capture the range of social cognitive strengths and weaknesses, an assessment battery may need to include multiple discourse genres (e.g., personal narratives, fictional retells, high-intimacy conversations, persuasion), vary in their elicitation procedures (e.g., providing/not providing visual stimuli), and elicit samples that are sufficiently long enough to “stress the system.” Within such a battery, both production and comprehension of MSTs should be assessed, and tasks specifically designed to assess social cognition, such as the *Video Social Inference Test* (Turkstra, 2008), *The Awareness of Social Inference Test-Short* (Honan et al., 2016; McDonald et al., 2017), Smarties task (Perner et al., 1989), Sally-Ann task (Baron-Cohen et al., 1985), and/or Strange Stories (Happé, 1994; White et al., 2009) (see Bosco et al., 2017 for use of the latter three tasks in participants with TBI) should be included. The battery should also include functional executive functioning measures, such as the FAVRES, to reveal the interplay between social cognition and executive functioning as well as their unique and combined impacts on psychosocial outcomes post-TBI. Additional research is needed to identify an assessment battery that can efficiently and accurately capture these cognitive-communication impairments observed post-TBI.

While many of the present study’s participants with TBI received treatment as usual, with individualized variations in duration and timing, details of the specific targets and treatments were not captured. Based on current findings, the following recommendations are made for designing treatments, though future controlled clinical trials are required to make any conclusive statements about treatments or their effects. Fictional narrative tasks, which appear to promote MST use, may be an appropriate place to start intervention targeting discourse and/or social cognition in adults with severe TBI. Increasing the length and elaboration of such stories, which tend to be reduced in the subacute stages of recovery (Greenslade et al., 2024), should also be a focus. With this focus, speakers may increase the raw number of MSTs used, without this skill being explicitly targeted. Following such success, intervention could move toward contextually and personally relevant narratives, as recommended by Steel et al. (2021), to promote skill generalization and maintenance and to target social cognitive abilities within discourse with high levels of intimacy/emotional investment (Byom and Turkstra, 2012). Given that individuals with TBI tend to make inaccurate judgments about the use of emotional MSTs in low vs. high intimacy conversations (Byom and Turkstra, 2012), the personal relevance of discourse may impact perspective-taking in this population; thus, it should be a consideration when designing intervention. Further, researchers and clinicians should acknowledge that speakers with lower educational attainment, more severe injuries (longer PTA), and/or lower verbal reasoning abilities may require direct intervention targeting MST use in discourse as well as social cognition in other contexts to maximize outcomes.

### 5.2 Limitations and future directions

While the present study’s findings offer critical insights into the social cognitive abilities of individuals post-TBI, several limitations must be acknowledged. First, this study compared Australian speakers with TBI and US speakers with NBI. Although the use of coding identification numbers was intended to keep coders naive to diagnosis (and timepoint), linguistic variations (e.g., more colloquial/informal lexical selection among Australian speakers compared to US speakers) could have biased some coding. That said, non-cultural factors, such as narrative length and quality, were equally likely to introduce bias. Beyond potential coding biases, we acknowledge that NBI speakers contributed only one sample, whereas longitudinal samples were collected from TBI speakers. Thus, future research should collect longitudinal NBI data to account for potential practice effects in the TBI group.

While the current study examined the diversity of MST use through the MST-TTR, it did not perform a micro-analysis to determine whether individuals with TBI may have overused common, nonspecific, and/or simple MSTs in contrast with more specific or advanced terms (Schwanenflugel et al., 1998; Armstrong, 2005). Future research should provide deeper analyses of the specific terms used in each population to determine whether the use of general MSTs may be an indicator of word finding difficulties in those with TBI. Analyses could also compare the relative use of cognitive, emotional, and desire terms across populations to determine if differences exist in the categories of MSTs being used by speakers with and without TBI. Further, analyses should compare the use of more advanced/conceptually complex MSTs across groups (Astington and Olson, 1990; Schwanenflugel et al., 1998).

Future research would also benefit from further exploration of MST use and understanding in adults with TBI. Existing research has explored MST use across several genres, including high and low intimacy conversations, persuasive discourse, and fictional narrative retells. Future research should apply the current coding scheme to personal narratives, such as the Important Event narratives from the TBIBank protocol. Applying this coding scheme would allow for separation of MST use as it relates to the speaker versus others in the narrative, which could provide additional insights into the effect of personal relevance/emotional investment on MST use in discourse for this population. Application to personal (versus fictional) narratives also has implications for the measure’s ecological validity and clinical applicability, given that personal narratives are more likely to affect the speaker’s personal, work, and community interactions, impacting psychosocial outcomes. In addition, research should further explore relationships between social acceptability perceptions of communication partners and/or lay observers and metrics of impoverished content and/or MST use across discourse genres. Although the nature of available brain scan data limited conclusions about relationships between MST use and the site of lesion(s) in the present study, analyses examining such relationships would also be informative. Moreover, given the mixed results from MST use in discourse post-TBI, future research also should investigate whether other measures, such as comprehension of MSTs and other metacognitive language, might more accurately identify social cognitive challenges in this population (Grazzani and Ornaghi, 2012).

Finally, to enhance the clinical utility of MST coding within discourse tasks, research should continue exploring the viability of automated speech recognition as a tool to improve the efficiency of transcription (Liu et al., 2023). From there, the current study’s MST lists compiled from Byom and Turkstra (2012), Bootsma et al. (2021), and Cinderella retells could be used to automatically identify these terms within discourse samples. Given that some terms have multiple meanings (e.g., “like” can be used as a filler or to convey desire as described in Bootsma et al., 2021), automatically identified terms would need to be confirmed as conveying a mental state. Yet, even with this additional step, automation could greatly improve the efficiency and reliability of MST analyses.

## 6 Conclusion

Overall, the present study found that familiar fictional narrative retells, such as *Cinderella*, may offer a facilitative context for observing the social cognitive abilities of adults post-TBI. No between-group differences or improvements in mental state term (MST) use were observed over the first two years post-TBI, after controlling for story length. Thus, impoverished discourse, rather than a specific deficit in MST use, was identified as negatively impacting social communicative exchanges. Although MST use was not impacted across all individuals with severe TBI, those who had lower levels of educational attainment, more severe injuries, and/or poorer verbal reasoning abilities post-TBI were more prone to show deficits in discourse-level MST use. Thus, these individuals may require direct treatment related to social cognition and discourse production to maximize their outcomes. Finally, clinicians and researchers should consider how differences in discourse genres, task demands, and personal relevance/emotional investment may impact the performance of individuals with TBI as they plan assessments and treatments for social cognition in this population. Accounting for these factors should enhance our development of effective assessment batteries and treatments, designed to improve relationships with family, friends, colleagues, and community members, with gains ultimately extending to quality of life.

## Data availability statement

Publicly available datasets were analyzed in this study. These data can be found here: TBIBank Togher corpus (doi:10.21415/T5R018), AphasiaBank control corpora contributed by Capilouto (doi:10.21415/HTMN-5P65), MSU (doi:10.21415/XBVQ-E342), Richardson (doi:10.21415/8KZF-5X33), and Wright (doi:10.21415/X12Y-GE35). Access to these clinical databases is password-protected and restricted to faculty members. Interested faculty members can gain access by following the guidelines at: https://talkbank.org/share/rules.html.

## Ethics statement

The studies involving humans were approved by Australian National Research Ethics Committee, East Carolina University UMCIRB, Montclair State University Institutional Review Board, University of Kentucky Office of Research Integrity, and University of New Mexico Institutional Review Board. The studies were conducted in accordance with the local legislation and institutional requirements. Written informed consent for participation in this study was provided by either the participants or their legal guardians/next of kin.

## Author contributions

KG: Writing – review & editing, Writing – original draft, Visualization, Validation, Supervision, Project administration, Methodology, Formal Analysis, Data curation, Conceptualization. CH: Writing – review & editing, Writing – original draft, Methodology, Formal analysis, Conceptualization. LH: Writing – review & editing, Writing – original draft, Methodology. LK: Writing – review & editing, Writing – original draft, Methodology. AR: Writing – review & editing, Writing – original draft. EB: Formal analysis, Writing – review & editing, Writing – original draft, Resources, Methodology, Investigation, Data curation, Conceptualization.

## Footnotes

^1^Because of inconsistencies in when the Glasgow Coma Scale was delivered (e.g., in the ambulance, after arriving at the hospital, after medication), these scores were used as part of inclusion criteria, but not as a predictor variable. Instead, PTA was selected as a predictor, reflecting injury severity.
